# The Rare Upper Thoracic and Cervical Esophageal GIST Resected Through Thoracoscopic Esophageal Mobilization and a Cervical Approach: A Case Report

**DOI:** 10.1002/cnr2.70423

**Published:** 2025-12-05

**Authors:** Kenjiro Ishii, Erica Nishimura, Yuki Tajima, Kumiko Hongo, Hiroto Fujisaki, Motohito Nakagawa, Kiminori Takano, Osahiko Hagiwara, Toshiyuki Enomoto, Takaharu Kiribayashi, Koji Asai, Takuya Nagata, Manabu Watanabe, Yoshihisa Saida

**Affiliations:** ^1^ Division of Surgery Toho University Ohashi Medical Center Tokyo Japan; ^2^ Division of Surgery Hiratsuka City Hospital Hiratsukashi Kanagawa Japan

**Keywords:** esophageal GIST, thoracoscopic surgery, upper thoracic esophagus

## Abstract

**Background:**

Gastrointestinal stromal tumor (GIST) is the most common mesenchymal neoplasm arising from the digestive tract; however, esophageal GIST is a very rare entity and represents < 1% of all GIST cases. Furthermore, esophageal GIST is most commonly located in the lower esophagus, while esophageal GIST in the upper esophagus is rare among them. Therefore there are very few reports regarding their resection methods.

**Case:**

This report describes a very rare resectable case of large upper esophageal GIST that extended to the cervical esophagus to be completely resected by performing thoracoscopic mobilization of the esophagus, followed by transection of the cervical esophagus via a cervical approach. The operative procedure was as follows: ensuring an adequate macroscopic margin for esophageal resection from within the thoracic cavity was judged to be difficult; therefore, only dissection of the thoracic and cervical esophagus was performed with thoracoscopic procedure, and the cervical esophagus was resected via a cervical approach. The abdominal procedure was carried out laparoscopically, and the specimen, including the tumor, was extracted from a small incision. Next, reconstruction using a gastric tube via a retrosternal approach was done. We also describe a literature review of upper esophageal GISTs.

**Conclusion:**

The surgical approach of large tumors of the upper thoracic and cervical esophageal GIST, including esophageal resection and reconstruction methods, needs to be carefully considered in advance. A cervical approach for esophageal resection is considered useful when it is difficult to resect the tumor's proximal end within the thoracic cavity safely.

## 1 Introduction

Gastrointestinal stromal tumor (GIST) is the most common mesenchymal neoplasm arising from the digestive tract and its annual incidence is 7–20 per million [1]. GIST can occur anywhere throughout the gastrointestinal tract, and arise primarily in the stomach (63%), followed by the small intestine (30%) and the colon or rectum (5%) [2]. Esophageal GIST is very rare and represents about 1%–2% of all cases [2, 3, 4]. Furthermore, esophageal GIST is most commonly located in the lower esophagus, while GIST in the upper esophagus is rare [5, 6]. As for the treatment, complete surgical resection is usually considered for localized GIST. Gastric and intestinal GISTs can be removed by segmental or wedge resection, while resection of esophageal GIST is limited to enucleation or esophagectomy because of its anatomical feature. To remove the esophageal GIST without damaging the membrane, esophagectomy is essentially needed. Esophagectomy is a very high invasive operation; therefore, enucleation can be a treatment option only when the tumor is small [7, 8].

The current case describes a very rare, large upper esophageal GIST extending to the cervical esophagus, which required a unique surgical strategy involving thoracoscopic mobilization and subsequent transection via a cervical approach, a method not previously reported for such a large and high‐lying GIST.

## 2 Case Presentation

A 70‐year‐old man came to a local hospital with nocturnal cough as the main complaint in January 2023. Chest to pelvis CT revealed a large esophageal tumor; therefore, the patient was referred to the gastroenterology department at Hiratsuka City Hospital (Kanagawa prefecture, Japan) in February 2023. As a result of endoscopic ultrasound‐fine needle biopsy (EUS‐FNB), the patient was diagnosed with a GIST of the upper thoracic esophagus, extending into the cervical area, and was referred to our department for surgical resection.

**FIGURE 1:**
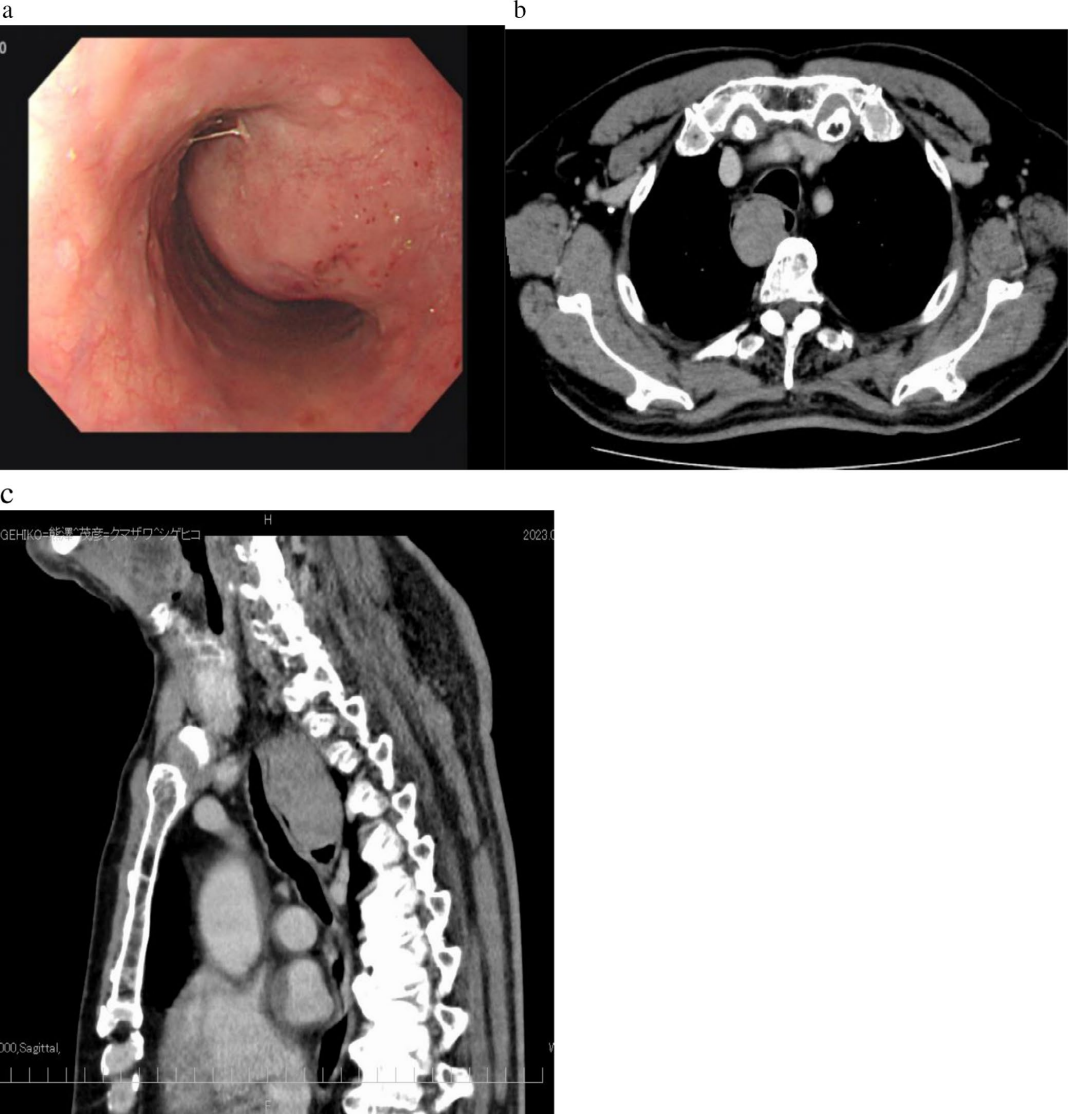
(a) Upper gastrointestinal endoscopy showing a large protruding submucosal lesion, approximately 50 mm in size, located in the upper thoracic esophagus between 21 and 26 cm from the incisor teeth. (b, c) Enhanced CT scans (coronal and axial views) demonstrating a well‐defined tumor with a maximum diameter of 55 mm. The mass is observed primarily in the upper thoracic esophagus, with clear extension into the cervical esophagus. CT showed a well‐defined tumor with a maximum diameter of 55 mm was observed, primarily in the upper thoracic esophagus, extending into the cervical esophagus.

Serum levels of carcinoembryonic antigen and carbohydrate antigen 19–9 were within normal range (CEA: 5.0 ng/mL or less, CA19‐9: 37 U/mL or less) Upper gastrointestinal endoscopy revealed a protruding lesion, 50 mm in size, in the upper thoracic esophagus between 21 and 26 cm from the incisor teeth. The edges were smooth, and the mucosal surface appeared normal, leading to a diagnosis of a submucosal tumor. CT showed a well‐defined tumor with a maximum diameter of 55 mm that was observed, primarily in the upper thoracic esophagus, extending into the cervical esophagus. No obvious lymph node enlargement was noted (Figure 1). EUS revealed a submucosal tumor, measuring 58.7 × 31.7 mm, originating from the esophageal muscular layer that was observed. The tumor exhibited a uniform hypoechoic area, with blood flow detected within the tumor. EUS‐FNB was done; the result was that proliferating spindle‐shaped cells were observed, and immunohistochemical staining results were as follows: SMA negative, desmin negative, c‐kit positive, CD34 positive, S100 negative, pan‐keratin (AE1/AE3) negative, and a Ki‐67 positivity rate was below 5%. Therefore, the submucosal tumor was diagnosed as esophageal GIST.

Given that the tumor is a large upper thoracic esophageal GIST extending into the cervical region, partial resection was considered to be difficult. Therefore, the plan is to proceed with a thoracoscopic esophagectomy with subtotal esophagectomy and reconstruction using a gastric tube via a retrosternal approach (April 2023).

**FIGURE 2:**
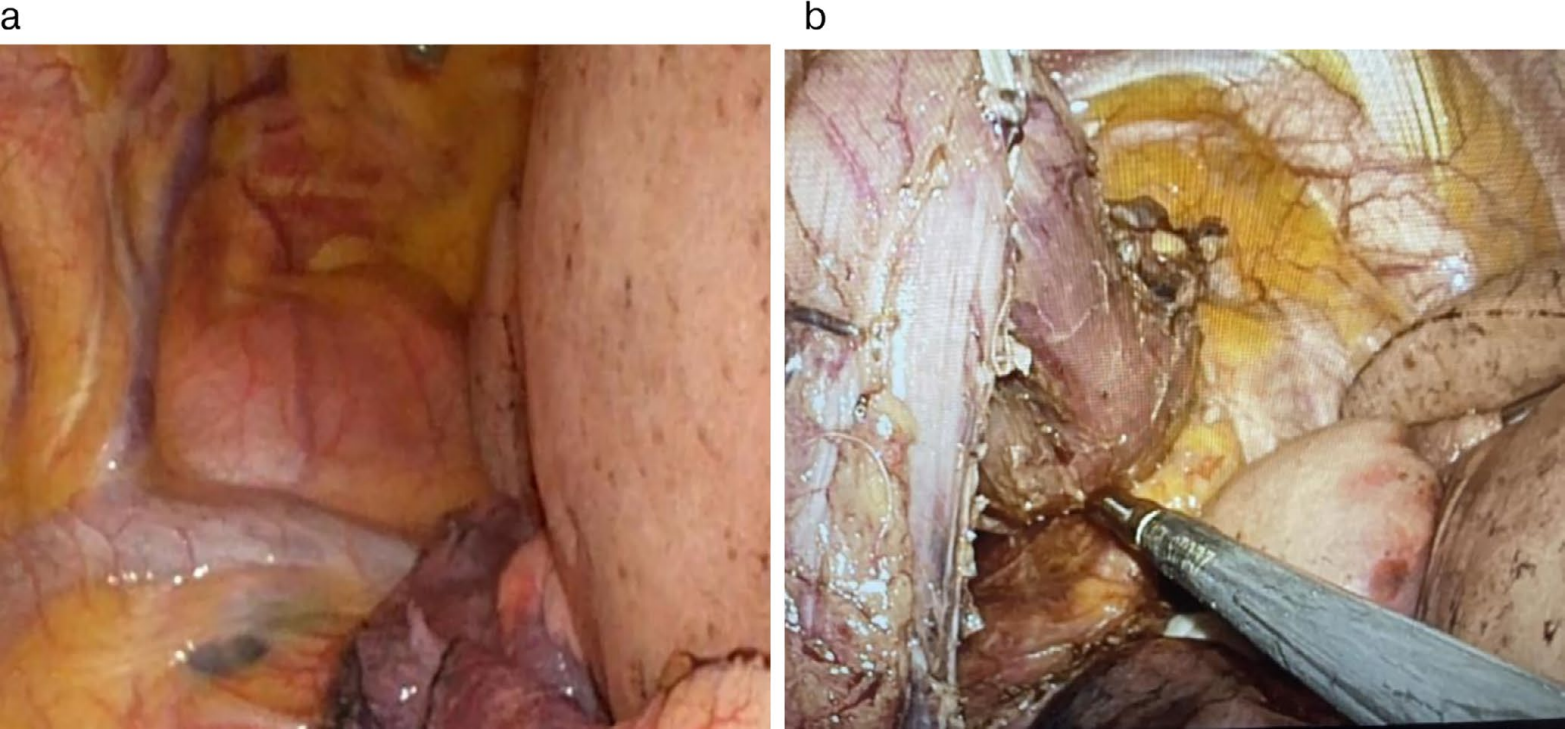
(a) Intraoperative thoracoscopic procedure for esophageal mobilization. View showing the difficulty in obtaining an adequate macroscopic proximal margin for esophageal transection from within the thoracic cavity due to the high location of the tumor. (b) Only mobilization and meticulous dissection of the thoracic and cervical esophagus, including the tumor‐bearing segment, were performed using the thoracoscopic procedure, ensuring the tumor pseudocapsule remained intact.

**FIGURE 3:**
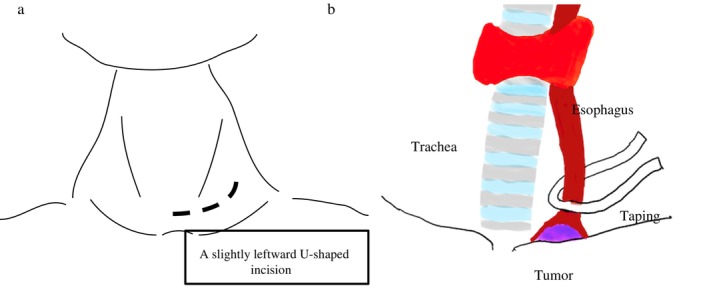
Cervical approach for proximal esophageal transection. (a, b) A slightly leftward U‐shaped incision was made for the cervical approach. The trachea was carefully retracted to the right, and meticulous dissection of the cervical esophagus was performed, paying close attention to the recurrent laryngeal nerve. The cervical esophagus on the oral side of the tumor was taped with Teflon tape. The cervical esophagus was then transected using a surgical stapler (autosuture device) above the tumor, securing the proximal margin.

The operative procedure was as follows; ensuring an adequate macroscopic margin for esophageal resection from within the thoracic cavity was judged to be difficult; therefore, only dissection of the thoracic and cervical esophagus, including the tumor, was performed with thoracoscopic procedure. The cervical esophagus on the oral side of the tumor was dissected and taped with Teflon tape. While applying traction to the tape, the cervical esophagus was transected using a surgical stapler (Figures 2 and 3). Next, the abdominal procedure was carried out laparoscopically. After ligating and dividing the vessels around the stomach, the esophagus was dissected from the diaphragmatic crura, and the tumor‐bearing esophagus was pulled into the abdominal cavity from the mediastinal side. The extraction was performed without any particular difficulty. The retrosternal space was made laparoscopically. A small incision was made in the upper midline of the abdomen, and a gastric tube was created outside the body, and the specimen, including the tumor, was extracted. Next, reconstruction using a gastric tube via a retrosternal approach was done. An ileostomy was created at the end. The total intraoperative blood loss was 100 mL, and the procedure was completed without any significant complications.

**FIGURE 4:**
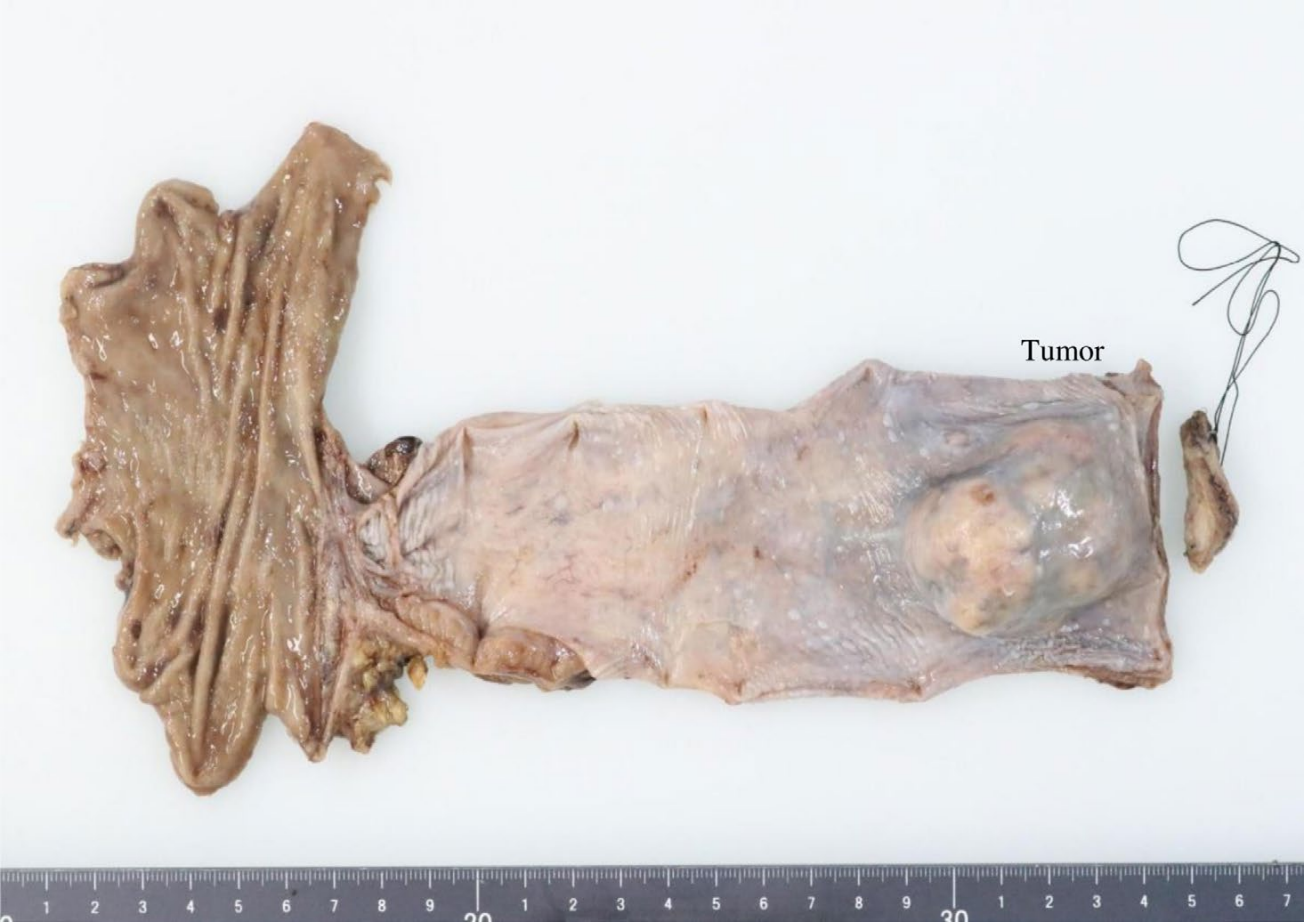
Pathological findings and tumor characteristics. (a) Macroscopic view of the excised specimen showing the large esophageal GIST (maximum diameter 52 mm). The tumor was completely excised, and the oral side stump was confirmed as negative, with no evidence of tumor pseudocapsule disruption. (b) Microscopic image (H&E or Immunohistochemistry not specified, but context suggests the tumor was c‐kit positive/GIST). The case was categorized as high risk according to the modified Fletcher classification.

The postoperative course was good, and there was no remarkable adverse event during the hospital stay. Postoperative management was conducted with a focus on enteral nutrition, coordinated by nutritionists [9]. Pathological findings were that the oral side stump was negative, with no evidence of tumor pseudocapsule disruption, and it was concluded that the tumor was completely excised. The maximum tumor diameter was 52 mm, and the esophagus was the site of origin (Figure 4). According to the modified Fletcher classification, this case was categorized as high risk (Figure 4).

Imatinib was introduced as adjuvant chemotherapy; however edema and fatigue were strong, so it was suspended soon. There has been no recurrence of GIST to date (June 2025, with CT scans every 6 months).

## 3 Discussion

Most esophageal stromal tumors are of smooth muscle origin, with approximately 70% being leiomyomas, and GISTs accounting for about 25% [10]. As stated above, esophageal GIST is rare and represents under 1% of all cases, and GIST in the upper esophagus is exceptionally rare among esophageal GISTs. According to previous reports, across the five major series summarized, only five cases out of a total of 218 esophageal GISTs were located in the upper esophagus (2.3%). This confirms that our case falls into a minority of highly challenging tumors [3, 5, 6, 11, 12, 13] (Table 1). The overwhelming prevalence of GISTs in the middle and lower esophagus suggests that most surgical experiences focus on those areas where standard thoracoscopic or abdominal/thoracoscopic approaches are feasible. The scarcity of upper esophageal cases highlights the lack of standardized, validated surgical approaches for the cervicothoracic junction, which is the most challenging location to achieve a clear proximal margin.

**TABLE 1:** A summary of previously published reports on esophageal GISTs.

Primary location	Lott et al. [5]	Feng et al. [6]	Kang et al. [3]	Kurosaki et al. [11]	Lian et al. [12]	Nakano et al. [13]	Total
Upper		2			3		5
Middle	4	13	3		12		42
Lower	46	99	15	2	8	1	171

In this case, the tumor was large and partially extended to the cervical area; therefore, enucleation was impossible and esophagectomy was needed to resect it without damaging the pseudocapsule. The most significant challenge was securing an adequate, safe proximal surgical margin for this high‐lying, large tumor without rupturing the pseudocapsule, a critical factor for recurrence‐free survival.

If the tumor is located in the middle or lower esophagus, it is typically possible to secure an adequate macroscopic margin and detach the oral side of the tumor during the thoracic procedure, which is the normal approach for thoracoscopic esophagectomy [3, 10]. However, in cases like the present one, where the tumor involves the upper esophagus extending into the cervical esophagus, this is exceedingly difficult and risky via the thoracic approach due to limited maneuverability and proximity to vital structures. Therefore, our alternative strategy was essential: the thoracic approach was limited to mobilization and dissection, and the cervical approach was used to safely achieve the proximal transection and secure a negative margin (oral side stump was negative). This two‐stage approach allowed for complete, safe oncological resection while utilizing the minimally invasive benefits of thoracoscopy. And finally, the tumor‐containing esophagus should be extracted from the abdomen.

In this specific case, we elected to proceed directly with surgical resection without neoadjuvant imatinib for the following critical reason: As mentioned above, the tumor was a large GIST (55 mm maximum diameter) located high in the upper thoracic esophagus, extending into the cervical esophagus. Even if imatinib therapy were to achieve a significant reduction in tumor size, the tumor would still necessitate a subtotal esophagectomy using the same hybrid approach (thoracoscopic mobilization followed by cervical transection) to safely secure the high proximal margin. Reducing the size of the tumor in this specific boundary area would not change the required extent of esophageal resection nor simplify the technical challenge of securing the oral surgical margin via the cervical approach. Therefore, since neoadjuvant therapy would not have altered the complexity of the required surgical approach, and to avoid delaying curative resection, we decided to proceed with upfront surgery. We believe this was a justified strategy for complete tumor excision with a clear margin.

The retrosternal route was selected for reconstruction in this case for the following two reasons: First, our surgical team has extensive technical familiarity with this route, which is our standard protocol for esophagectomy. Second, as this high‐risk GIST necessitates long‐term surveillance, locating the conduit via the retrosternal route prevents potential local recurrence from directly narrowing the reconstructed esophagus.

Thoracoscopic surgery for esophageal GISTs is considered useful from the perspective of minimizing postoperative wound pain, respiratory dysfunction, and cosmetic invasiveness due to chest wall disruption. Furthermore, it is recommended that robotic esophagectomy be considered for this type of procedure, which offers the opportunity for delicate and precise maneuvers [14]. However, limitations in maneuverability with forceps and the emergence of blind spots due to the magnified field can occur; therefore special caution is needed, especially in cases such as GIST, where careful removal without damaging the pseudocapsule is required. It is considered important to have a detailed surgical plan prior to the procedure.

## 4 Conclusion

Large tumors of the upper thoracic and cervical esophageal GIST are extremely rare, and the surgical approach, including esophageal resection and reconstruction methods, needs to be carefully considered in advance. The peculiarity of this case—a large GIST involving the high upper thoracic and cervical esophagus—demonstrates that the standard thoracoscopic esophagectomy technique may be inadequate for safe oncological clearance of the proximal margin. The key lesson to be drawn is that a cervical approach for esophageal resection is considered useful and, in some cases, necessary when it is difficult to safely resect the tumor's proximal end within the thoracic cavity, allowing for complete resection and margin control.

## Author Contributions

Kenjiro Ishii designed the study. Kenjiro Ishii analyzed the data and wrote the manuscript. Kenjiro Ishii, Erica Nishimura, and Yuki Tajima collected and collated the clinical data. Kumiko Hongo, Hiroto Fujisaki, Motohito Nakagawa, Kiminori Takano, Osahiko Hagiwara, Toshiyuki Enomoto, Takaharu Kiribayashi, Koji Asai, Takuya Nagata, Manabu Watanabe, and Yoshihisa Saida reviewed the manuscript. All authors have read and agreed to the published version of the manuscript.

## Ethics Statement

All authors of this article certify that this work was conducted and submitted in accordance with the Committee on Publication Ethics and Wiley's Publication Ethics guidelines.

## Consent

A statement of written informed consent has been obtained from the patient for the publication of case details and use of any images.

## Conflicts of Interest

The authors declare no conflicts of interest.

## Data Availability

The data that support the findings of this study are available from the corresponding author upon reasonable request.
